# Patient Satisfaction after Upper Extremity Laser Lipolysis without Suction

**DOI:** 10.1155/2011/352451

**Published:** 2011-09-22

**Authors:** Brett S. Kotlus, Charles Mok

**Affiliations:** Allure Medical Spa, 8180 26 Mile Road, Suite 300, Shelby Township, MI 48316, USA

## Abstract

*Background and Objective*. There has been a heightened interest in laser-assisted fat reduction procedures. We aimed to determine if lipolysis with the 1,320 nm Nd-YAG short-pulsed laser without subsequent suction results in satisfactory contouring of the upper extremity. *Materials and Methods*. Unilateral laser lipolysis of the upper arm was performed on 5 patients. Subcutaneous, subdermal, and skin surface temperatures were monitored with flexible thermocouples throughout the procedure to aid in the establishment of a treatment endpoint. Photographs and arm circumference measurements were evaluated before and 3 months after laser lipolysis. Patients were given the choice of undergoing the procedure on the contralateral arm at 3 months. *Results*. All patients achieved no improvement to minimal improvement in upper arm contour. One of five patients was elected to have lipolysis performed on the contralateral arm. *Conclusion*. Laser lipolysis may be safely performed with the parameters utilized in this pilot study, although minimal improvement was seen in upper extremity contour.

## 1. Introduction

A number of fat-reduction procedures have emerged over recent years to address a demand for less invasive approaches, with an ultimate goal of imparting less risk while producing favorable results. These include the use of tumescent solutions [1], smaller gauge cannulas [2], external ultrasound [3], low-level external laser [4], injection lipolysis [5, 6], cryolipolysis [7], external radiofrequency energy [8], and percutaneous laser lipolysis [9].

Laser lipolysis has been described with and without concurrent suction lipectomy [9, 10]. Laser energy passes through an optical fiber and is directed at the subcutaneous fat layer. Adipocyte damage, or more specifically adipocyte cell membrane damage, can lead to cell content leakage to the interstitial compartment with potential irreversible cell collapse. Suction applied to the area can clear the interstitial fatty debris or this material can be allowed to be handled by innate metabolic mechanisms.

The current pilot study aimed to evaluate patient satisfaction after laser lipolysis without suction of the proximal upper extremity with a treatment endpoint of 41 degrees Celsius in the subcutaneous layer. This temperature was selected based on previous determinations that higher sustained dermal temperatures can lead to irreversible necrosis [11].

## 2. Materials and Methods

Five female patients who expressed a desire for improved upper arm contour were included in this prospective pilot study which was performed according to the guidelines of the 1975 Helsinki Declaration. Subjects with a history of previous cosmetic upper extremity procedures or striae distensae in the area of interest were excluded.

The mean subject age was 35 years (range: 28–45 years), and the mean weight was 78.2 kilograms. Subjects were randomly assigned to right-or left-sided treatment. They were offered the option of contralateral treatment to be performed three months after the initial procedure. Standardized photographs and arm circumference measured midway between the olecranon and the midaxillary crease were obtained before the procedure and three months after the procedure. Satisfaction surveys were performed at three months. Subjects were asked if there was no change, minimal change, or substantial change in the treatment area as compared to the untreated side.

**Figure 1:**
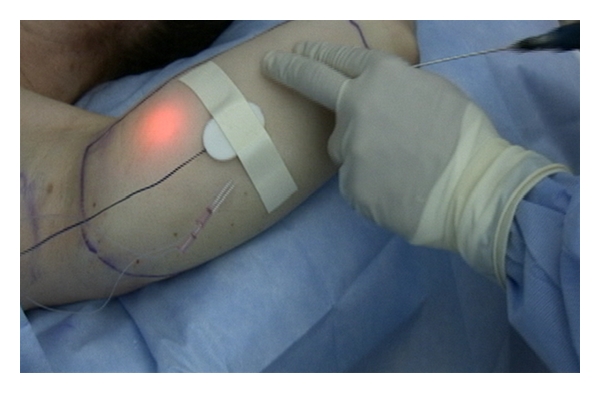
1,320 nm laser lipolysis of the proximal upper extremity.

**Figure 2:**
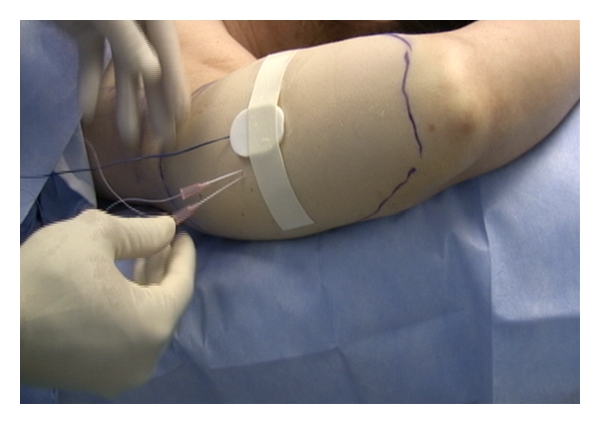
Placement of thermocouples.

Approximately 250 milliliters of a warmed buffered tumescent solution containing 0.05% lidocaine and 1 : 1,000,000 epinephrine was infiltrated subcutaneously twenty minutes before the procedure. Lipolysis was performed with a 1,320 nm Nd:YAG pulsed laser (CoolLipo, CoolTouch Inc., Roseville, CA, USA) at 15 Watts, 30 Hertz, and a pulse width of 100 *μ*sec. Energy was delivered subcutaneously via a 1.5-millimeter cannula housing a 500-micrometer fiberoptic extending 3 millimeters beyond the cannula tip. Entry was obtained through an elbow crease with a 1-millimeter skin punch, and cannula passes were performed with multiple passes at varied depths. Illumination of the fiber tip with an aiming beam was observed throughout the procedure, indicating the relative cannula depth (Figure 1). Thermocouple sensors (Thermes USB, Physitemp Instruments Inc., Clifton, NJ, USA) were placed at the subcutaneous, immediate subdermal, and skin surface levels (Figure 2). The treatment endpoint was when a subcutaneous temperature of 41 degrees Celsius was reached as measured with digital recording system (DasyLab, Measurement Computing, Norton, MA, USA).

At the conclusion of the lipolysis treatment, fluid and liquefied fat was manually expressed from the adit, site but suction lipectomy was not performed. Subjects were placed in an elastic postprocedural compression garment for 1 week.

## 3. Results

All five subjects completed the pilot study. Two subjects experienced mild ecchymosis, four subjects experienced mild to moderate soreness, and all subjects experienced mild edema at the treatment site. No other complications were noted. The mean total energy delivered per subject was 13,634 joules (range: 8,524 joules–21,242 joules). The target endpoint of 41 degrees Celsius in the subcutaneous layer was achieved in all treatments (Figure 2).

**Figure 3:**
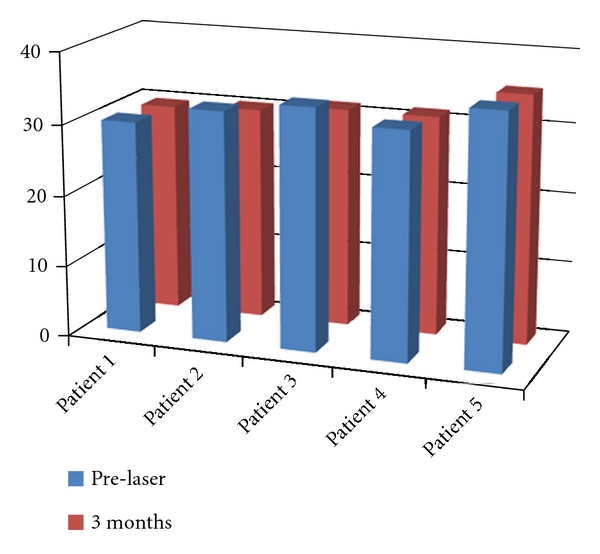
Arm circumference (in centimeters) before and after laser lipolysis.

**Figure 4:**
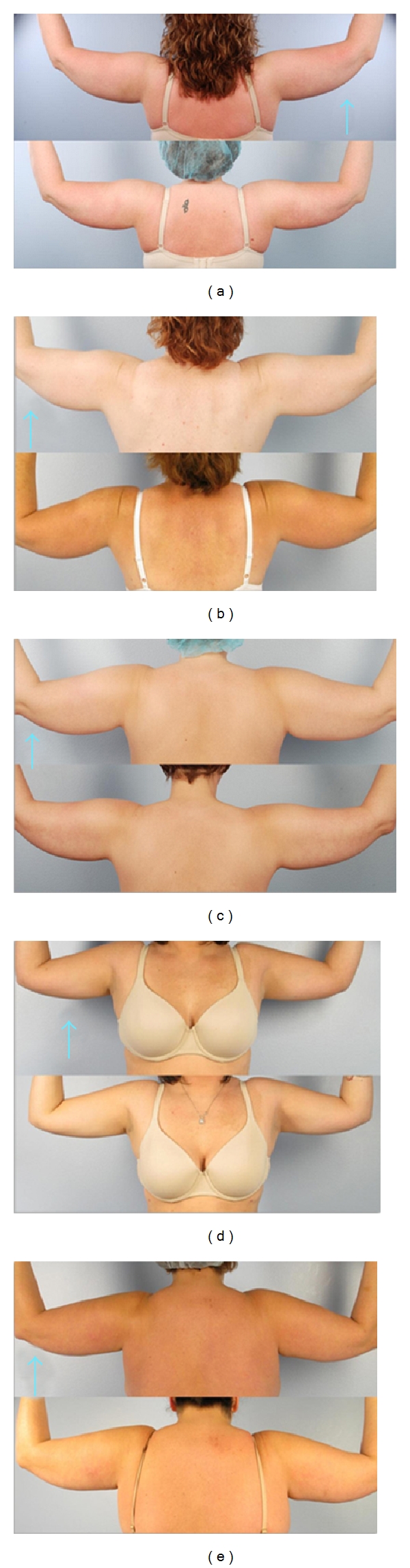
Before and after laser lipolysis (arrow indicates treated arm).

The mean change in midarm circumference three months after treatment was 0 centimeters ±1.8 (range −1.5–2 centimeters) (Figure 3). No observable improvement was noted in the treated arms in comparison photos at three months by independent observer evaluation (Figure 4). 

Three of five subjects reported minimal change and two of five subjects reported no change in the treated arm as compared to the contralateral side at three months. One of five subjects was elected to have contralateral laser lipolysis treatment. This subject received the highest total treatment energy (21,242 joules).

## 4. Discussion

There is currently no consensus on accepted treatment guidelines for body contouring with laser lipolysis. Considerations include optimization of wavelength [11, 12], energy settings, and treatment endpoints. The parameters used in this small pilot study did not lead to high patient satisfaction.

Dudelzak et al. reported a reduction in arm circumference in a series of 20 subjects in which 10 underwent 1,064 nm laser lipolysis without suction [10]. These patients received 7,080–12,026 J of energy during treatment. They also reported skin retraction and tightening in 16 subjects, but they did not describe how this was measured. In the present study, similar energy levels were delivered, but size reduction was not observed.

Kim and Geronemus described laser lipolysis without suction in the submental region [9], revealing more substantial fat reduction occurring in subjects receiving the highest cumulative energy in the series. 

In our series, the patient requesting contralateral treatment had the highest cumulative energy. Higher power settings or longer treatment times while maintaining safe temperature levels could potentially improve the results seen here. Ultimately, algorithms may be developed to aid in selecting the total energy required to treat a given fat volume, based on preprocedural subcutaneous thickness and surface area measurements.

The tumescent technique, as applied in this study, has been shown to promote a safe and relatively painless procedure [13]. Tumescent fluid, even when heated to body temperature, acts as a heat sink as laser energy is transmitted to the treatment zone. As such, the dermis is somewhat more protected from inadvertent burns when heat is applied to the subcutaneous tissue. At the same time, more energy may be needed to produce desired results than if no heat sink were present. 

**Figure 5:**
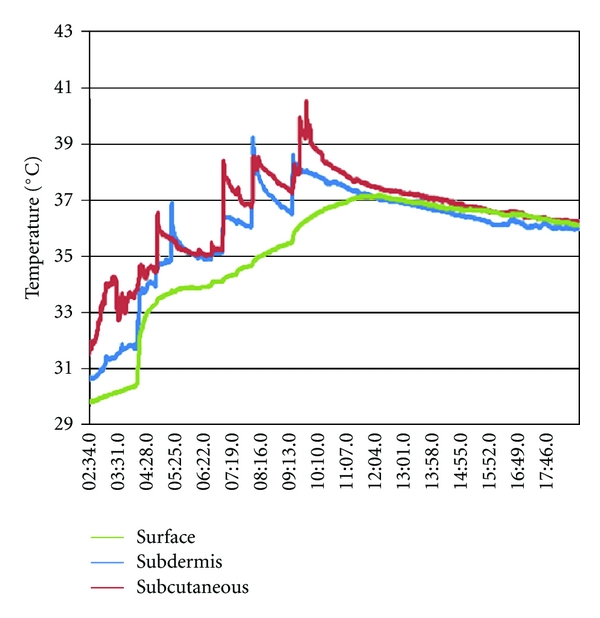
Thermocouple measurements during laser lipolysis treatment at 3 depths.

There was a 2–4 degree temperature gradient between subcutaneous and surface temperature as measured by thermocouples (Figure 5). A higher gradient would likely be seen where a greater thickness of subcutaneous fat is present, and this should be taken into account when selecting a treatment endpoint.

Some practitioners advocate laser application to the immediate subdermal plane with the goal of stimulating skin contraction [14]. The current study did not investigate this aspect of subcutaneous laser treatment as the subjects had satisfactory skin turgor. In such cases, suction lipectomy with resultant subcutaneous reshaping generally leads to skin accommodation, without the need for further skin tightening [15]. It was assumed that in the same way, laser lipolysis would afford sufficient skin redistribution.

There is a current overwhelming availability of devices that deliver laser energy to subcutaneous tissues. This market supply is driven in part by patients that actively seek aesthetic procedures that employ laser technology and that promise less invasive methods. At the same time, aesthetic physicians wish to satisfy patient desires and improve results while maintaining safety, reliability, and reproducibility. Further clarification through directed investigations will help to clarify the role of lasers in fat modification.
